# Learning Environment, Preparedness and Satisfaction in Osteopathy in Europe: The PreSS Study

**DOI:** 10.1371/journal.pone.0129904

**Published:** 2015-06-23

**Authors:** Emanuele Luciani, Patrick L. S. van Dun, Jorge Eduardo Esteves, Christian Lunghi, Marco Petracca, Liria Papa, Olivier Merdy, Anne Jäkel, Francesco Cerritelli

**Affiliations:** 1 Clinical-based Human Research Department, Research Division, C.O.ME. Collaboration, Pescara, Italy; 2 Research Division, C.O.ME. Collaboration, Mechelen, Belgium; 3 Free University of Brussels, Department of Osteopathic Sciences, Brussels, Belgium; 4 British School of Osteopathy (BSO), London, United Kingdom; 5 Centro Ricerche Olistiche per la Medicina Osteopatica e Naturale (CROMON), Rome, Italy; 6 Centre pour l'Etude, la Recherche et la Diffusion Osteopathiques (CERDO), Rome, Italy; 7 International College of Osteopathic Medicine (ICOM), Milan, Italy; 8 Institut des Hautes Etudes Ostéopathiques (IdHEO), Orvault, France; 9 European School of Osteopathy (ESO), Maidstone, United Kingdom; National Institute of Health, ITALY

## Abstract

**Objective:**

1) to assess the preparedness to practice and satisfaction in learning environment amongst new graduates from European osteopathic institutions; 2) to compare the results of preparedness to practice and satisfaction in learning environment between and within countries where osteopathy is regulated and where regulation is still to be achieved; 3) to identify possible correlations between learning environment and preparedness to practice.

**Method:**

Osteopathic education providers of full-time education located in Europe were enrolled, and their final year students were contacted to complete a survey. Measures used were: Dundee Ready Educational Environment Measure (DREEM), the Association of American Medical Colleges (AAMC) and a demographic questionnaire. Scores were compared across institutions using one-way ANOVA and generalised linear model.

**Results:**

Nine European osteopathic education institutions participated in the study (4 located in Italy, 2 in the UK, 1 in France, 1 in Belgium and 1 in the Netherlands) and 243 (77%) of their final-year students completed the survey. The DREEM total score mean was 121.4 (SEM: 1.66) whilst the AAMC was 17.58 (SEM:0.35). A generalised linear model found a significant association between not-regulated countries and total score as well as subscales DREEM scores (p<0.001). Learning environment and preparedness to practice were significantly positively correlated (r=0.76; p<0.01).

**Discussion:**

A perceived higher level of preparedness and satisfaction was found amongst students from osteopathic institutions located in countries without regulation compared to those located in countries where osteopathy is regulated; however, all institutions obtained a ‘more positive than negative’ result. Moreover, in general, cohorts with fewer than 20 students scored significantly higher compared to larger student cohorts. Finally, an overall positive correlation between students’ preparedness and satisfaction were found across all institutions recruited.

## Introduction

Osteopathy is a primary contact healthcare profession, which is becoming more popular worldwide and in particular in Europe. With an increasing number of patients and professionals moving around Europe to live, study and work, several international organisations and authorities are committed to develop and promote best osteopathic practice and training, so that patients can be safeguarded wherever they are [1]. In 2010, the World Health Organization published a benchmark document for training in osteopathy, which serves as a reference for national authorities wishing to establish systems of professional education and training, examination and licensure in osteopathy [2]. This document was particularly timed because in recent times there has been a significant increase in the number of universities and specialist colleges offering training in osteopathy worldwide, and an associated increase in the number of students attending these courses; in parallel, the regulation of providers has been increasing [3].

Despite this promising picture, the standards of professional education, training and practice in osteopathy vary significantly across different countries. Consequently, in Europe, the osteopathic academic community has been actively involved in creating a common ground of professional training standards across countries, despite the heterogeneous political and bureaucratic situation [4]. Similarly, the Forum for Osteopathic Regulation in Europe (FORE) has established a series of priorities and guidelines to support the development and implementation of high quality osteopathic specific higher education degree programmes in Europe; to help standardise education standards between institutions; and to define the professional capabilities that students are required to achieve at the point of graduation [5]. Notwithstanding these initiatives, their legal basis is limited and they are not designed to override national law; Europe still lacks of an effective regulatory framework to deliver high standards of osteopathic care and training. This scenario is further aggravated by the fact that in Europe, the regulation of osteopathy as a profession is limited to eight countries, i.e., Finland, France, Iceland, Lichtenstein, Malta, Portugal, Switzerland and the UK; elsewhere the statutory regulatory process is still ongoing [6].

One of the aims of medical education is to ensure that graduates are prepared for autonomous clinical practice, therefore equipped with the knowledge, skills and personal and professional capabilities to work safely [7, 8]. According to Burford and colleagues (2014, p.1) “preparedness also implies that they themselves are aware for their capabilities, and are confident in their ability to safely begin work”. Notwithstanding this, differences in preparedness for practice were observed between different universities and programmes [9–11], thus suggesting that the educational environment may be responsible for it [10, 12]. Importantly, Brown and colleagues [13] have argued that the level of practitioner's competence is not simply a reflection of the educational institution from where they graduated. Varied levels of preparedness to practice have a critical impact on the clinical effectiveness and safety of patient care; however, “it is very difficult to assess the impact of graduates preparedness on patient care” ([14] pp.13).

Within the context of osteopathy, the level of preparedness to practice has been recently investigated. Freeth and co-workers (2012) found that although UK osteopathy graduates were safe to practice, they lacked capabilities in business and practice management skills, patient management and interpersonal communication capabilities [15]. More recently, Luciani and colleagues (2014) found that perceived preparedness was higher in a French osteopathic institution than in one Italian and one British institution; despite this, the overall preparedness scores were positive in all institutions [16]. Similarly, Vaughan et al. (2014) found positive scores among two Australian institutions [17]. Also in Australia, Subramaniam and colleagues (2014) [18] found that students perceived a lack of competence in several clinical areas, and their lack of preparedness was linked to increased stress during the transition from student to practitioner [19, 20]; this may negatively impact on the effectiveness and safety of osteopathic care.

Evaluating the educational environment is a critical component of academic quality assurance, curriculum development and students' satisfaction [13, 17]. Currently, there is a scarcity of studies investigating satisfaction, learning environment and preparedness for practice in the field of osteopathy [16, 17]. Importantly, no research has been conducted on a large cohort of European osteopathic academic and professional institutions to investigate students’ satisfaction and level of preparedness to practice. To this end, the general objectives of the present cross-sectional study were: 1) to assess the preparedness to practice and satisfaction in learning environment in new graduates from European osteopathic education institutions; 2) to compare the results of preparedness to practice and satisfaction in learning environment between and within countries where osteopathy is statutory regulated and where regulation is still to be achieved; 3) to identify possible correlations between learning environment and preparedness to practice.

## Materials and Methods

### Setting

The study was carried out in providers of full time education and training in osteopathy based in Europe. Typically, length of full time education ranges from 4 to 6 years, according to the European country and programme considered.

### Participants

The sample consisted of 314 students attending the final weeks of semester 2 in the academic year 2013/2014 in 8 private and 1 public institution. Participants were contacted by email by the contact at each institution.

### Instrument

A short demographic questionnaire was constructed to collect information such as participant's age, gender and previous healthcare experience. Learning environment was assessed using the Dundee Ready Educational Environment Measure (DREEM) questionnaire and perceived preparedness was assessed using the Association of American Medical Colleges (AAMC) questionnaire, both were rated via 5-point Likert scale (strongly agree = 4, agree = 3, uncertain = 2, disagree = 1, strongly disagree = 0); higher scores equate to better learning environments and levels of preparedness. Both questionnaires were used in a previous study [16]. The DREEM questionnaire has been used worldwide to assess the learning environment and is a validated and reliable inventory [17, 21]. It consists of 50 statements and participants are asked to select a response. Items 4,8,9,17,25,35,39,48 and 50 are negatively worded. The 50 items are divided into five subscales: Students' Perception of Learning (SPL), Students' Perception of Teachers (SPT), Students' Academic Self-Perception (SASP), Students' Perception of Atmosphere (SPA) and Students' Social Self-Perception (SSSP). The AAMC consists of 7 statements and was used to assess the perceived preparedness in few other studies [12, 16]. The domains represented a wide range of competencies in seven clinical areas, summarised as follows: 1) general clinical skills, 2) basic knowledge of diagnosis and management of common conditions, 3) communication skills, 4) skills for applying clinical decision making and evidence-based medicine to clinical care, 5) basic abilities on managing issues in medicine, 6) professionalism, and 7) basic abilities for patient care. Participants rated each of these areas using a five-point Likert scale: 1 (strongly agree), 2 (agree), 3 (neither agree nor disagree), 4 (disagree), and 5 (strongly disagree).

The Italian version of the questionnaires was used for institutions located in Italy. The French version was adopted for schools based in France and Belgium; both versions were utilised in a previous study [16]. The English version was administered to institutions where the osteopathy programme was taught in English language and also in the osteopathic education institution in the Netherlands.

### Procedures

The Centre for Osteopathic Medicine Collaboration (COME) was the promoter of the study and was in charge of controlling all procedures. 25 researchers, representing each European osteopathic education institution, were contacted by email and asked to participate with their institution. 12 responded positively and were informed regarding study details and procedures as well as timeline to enrol students. 3 institutions dropped out due to unavailability to fulfil the study procedures. Thus, 9 European institutions were finally enrolled: 1) BSO: British School of Osteopathy, London, UK, private institution; 2) ESO: European School of Osteopathy, Maidstone, UK, private; 3) IdHEO: Institut des Hautes Etudes Ostéopathiques, Orvault, France, private; 4) ULB: Université Libre de Bruxelles, Brussels, Belgium, public; 5) SC: Sutherland College, Amsterdam, The Netherlands, private; 6) ICOM: International College of Osteopathic Medicine, Milan, Italy, private; 7) CROMON: Centro Ricerche Olistiche per la Medicina Osteopatica e Naturale, Rome, Italy, private; 8) CERDO: Centre pour l'Etude, la Recherche et la Diffusion Osteopathiques, Rome, Italy, private; 9) AIOT: Accademia Italiana Osteopatia Tradizionale, Pescara, Italy, private.

After enrolment, students were informed about the study by the representing researcher and a recruiting advert was posted. Participants received an explanatory statement detailing the study and confidentiality as well as anonymity were assured. Consent for participation was inferred by their completion of the questionnaire.

All questionnaires and the participant information sheet were uploaded into an ad-hoc online platform already used in a previous study [16] and only final year students attending the last semester were able to access it. For this reason, a personalised password and username was created to log into the platform. A reminder was sent three months later and the on-line access was closed four months after the initial invitation. In this study, students could leave their own email allowing the researchers to follow up with a further survey after one year. Ethical approval for the study was granted by the AIOT Research and Ethics Committee.

### Statistical analyses

Statistical analyses were performed using mean, standard deviation, standard error of the mean with 95% confidence interval for continuous variable and point estimates for categorical variables. Assessment of normality was performed using robust Brown-Forsythe Levene-type test. According to Roff et al., the DREEM scores were presented by item along with the total and subscales scores [21]. Statistics presented considered: validation, measured by internal consistency of the DREEM using Cronbach’s alpha for both the subscale and total scores, as well as stability of the DREEM scores using Pearson’s *r*. Item-to-total correlation was also calculated. Comparison between schools were computed using one-way ANOVA to explore whether any difference in the DREEM and AAMC exists taking into account gender, country, age and previous experience. Effect size calculations (Cohen’s *d*) were shown where appropriate. Chi-square test was carried out to indicate any association between categorical data. *t*-test was used to test any difference between regulated vs not-regulated countries. Generalized linear model was also performed to determine any correlation between the DREEM and the AAMC. Missing data was handled using multiple imputation techniques and sensitivity analysis was applied to explore any resulting differences. Level of significance was set at 0.05. Analyses were performed using R statistical program v3.1.2 [22].

## Results

**Fig 1:**
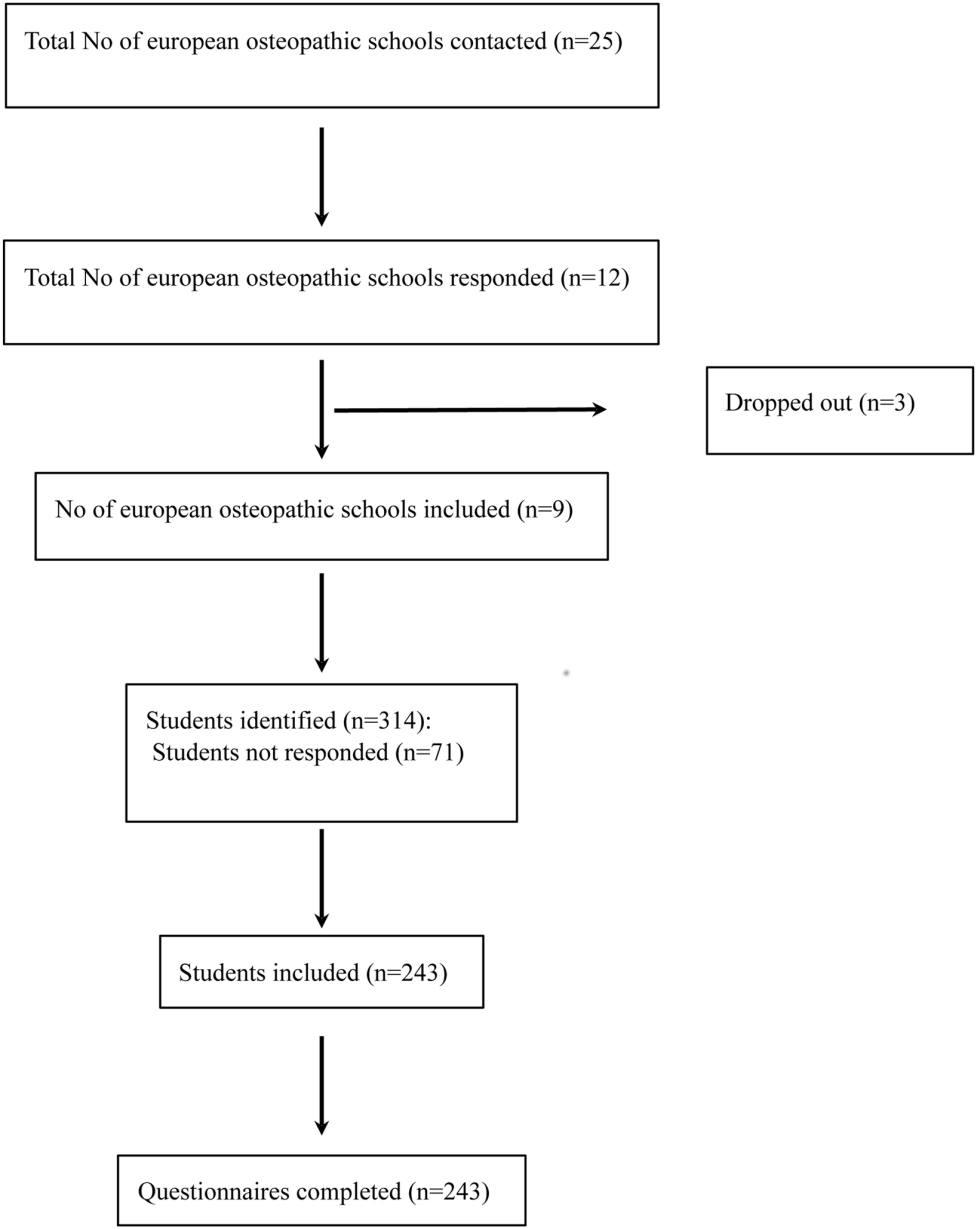
Flowchart of the institutions and students enrolled in the study.

243 final year students (77%) completed the survey (Fig 1). No blank questionnaires were received. The response rates by institution were as follows (see S1 Table): British School of Osteopathy (BSO: 75/90, 83%), European School of Osteopathy (ESO: 44/60, 73%), Institut des Hautes Etudes Ostéopathiques (IdHEO: 39/61, 64%), Université Libre de Bruxelles (ULB: 14/19, 74%), Sutherland College (SC: 16/20, 80%), International College of Osteopathic Medicine (ICOM: 20/25, 80%), Centro Ricerche Olistiche per la Medicina Osteopatica e Naturale (CROMON: 13/14, 93%), Centre pour l'Etude, la Recherche et la Diffusion Osteopathiques (CERDO: 12/15, 80%) and AIOT (10/10, 100%). Socio-demographic data of the study sample are presented in Table 1. No statistically significant differences were found between institutions.

**Table 1:** General characteristics of the study population.

Institutions		BSO	ESO	IdHEO	ULB	SC	AIOT	CROMON	CERDO	ICOM
Number of students		(n = 75)	(n = 44)	(n = 39)	(n = 14)	(n = 16)	(n = 10)	(n = 13)	(n = 12)	(n = 20)
Gender*	**Male**	39(52.0)	23(52.3)	13(33.3)	6(42.9)	8(50.0)	7(70.0)	4(30.8)	6(50.0)	10(50.0)
	**Female**	36(48.0)	21(47.8)	26(66.7)	8(57.1)	8(50.0)	3(30.0)	9(69.2)	6(50.0)	10(50.0)
**Age §**		27.3(0.4)	28.4(0.5)	25.3(0.3)	24.9(0.5)	28.3(0.8)	23.7(0.3)	25.6(1.5)	25.5(0.6)	24.9(0.3)
Previous experience*	**Yes**	33(44.0)	22(50.0)	9(23.1)	12(85.7)	7(43.8)	0 (0.0)	2(15.4)	1(8.3)	3(15.0)
	**No**	42(56.0)	22(50.0)	30(76.9)	2(14.3)	9(56.2)	10(100.)	11(84.6)	11(91.7)	17(85.0)

* N (%).

^§^ mean (±SEM).

BSO: British School of Osteopathy; ESO: European School of Osteopathy; IdHEO: Institut des Hautes Etudes Ostéopathiques; ULB: ULB University; SC: Sutherland College; ICOM: International College of Osteopathic Medicine; CROMON: Centro Ricerche Olistiche per la Medicina Osteopatica e Naturale; CERDO: Centre pour l'Etude, la Recherche et la Diffusion Osteopathiques; AIOT: Accademia Italiana Osteopatia Tradizionale.

### Validity and psychometric properties

With regard to validity, high internal consistency (Cronbach’s alpha > 0.70) was found considering the total and subscale DREEM scores (S2 Table). Similar results were found if item-to-total scores were concerned (S3 Table).

### DREEM and AAMC scores

Descriptive statistics were used to report DREEM and AAMC data (Table 2). The DREEM total score mean was 121.4 (SEM: 1.66) whilst the AAMC was 17.6 (SEM: 0.35). Considering institutions, the DREEM range ranged from 105.3 to 149.9, while the AAMC ranged from 14.7 to 22.9. This can be interpreted as all institutions achieved a ‘more positive than negative’ overall DREEM score (Table 3). The DREEM sub-scores were similar across institutions except for Students’ Perception of Teachers (SPT) where the BSO scored ‘in need of some retraining’ and Students’ Academic Self-Perception (SASP) that showed heterogeneous results. In fact, two institutions (BSO and ESO) scored as ‘many negative aspects’ whereas CROMON obtained the highest result: ‘Confident’. All other schools achieved the ‘Feeling more on the positive side’ score (Table 4).

**Table 2:** DREEM scores and AAMC overall score according to schools.

Questionnaire	BSO	ESO	IdHEO	ULB	SC	AIOT	CROMON	CERDO	ICOM
**DREEM Overall score**	105.3(1.6)	108.8(3.5)	129.4(3.9)	133.0(6.8)	149.9(3.4)	140.9(7.6)	149.8(4.1)	124.8(8.5)	131.8(5.3)
**SPL**	27.4(0.5)	25.4(1.0)	30.0(1.0)	31.0(1.6)	34.9(1.0)	33.2(1.9)	35.3(1.1)	30.3(2.3)	31.1(1.5)
**SPT**	22.0(0.6)	24.5(0.9)	27.3(0.9)	26.9(2.3)	32.5(0.9)	29.4(2.5)	32.2(1.3)	25.9(2.3)	28.4(1.4)
**SASP**	16.7(0.6)	16.0(0.8)	21.2(0.8)	23.6(1.1)	24.0(0.9)	24.5(1.6)	26.1(1.2)	21.2(1.1)	23.6(1.0)
**SPA**	24.9(0.6)	26.6(1.0)	32.3(1.0)	31.9(1.9)	37.7(0.9)	33.2(1.9)	35.7(1.1)	28.9(2.5)	30.8(1.5)
**SSSP**	14.4(0.4)	16.4(0.6)	18.7(0.7)	19.6(0.8)	20.9(0.5)	20.6(1.0)	20.5(0.7)	18.4(1.1)	18.1(0.8)
**AAMC Overall score**	14.7(0.5)	15.6(0.7)	18.6(0.9)	20.1(1.4)	21.4(0.6)	21.3(1.8)	22.9(1.0)	16.8(1.5)	21.0(1.2)

Numbers are expressed in mean (±SEM). BSO: British School of Osteopathy; ESO: European School of Osteopathy; IdHEO: Institut des Hautes Etudes Ostéopathiques; ULB: ULB University; SC: Sutherland College; ICOM: International College of Osteopathic Medicine; CROMON: Centro Ricerche Olistiche per la Medicina Osteopatica e Naturale; CERDO: Centre pour l'Etude, la Recherche et la Diffusion Osteopathiques; AIOT: Accademia Italiana Osteopatia Tradizionale.

**Table 3:** DREEM scores range for general interpretation.

Overall DREEM score	Interpretation
0–50	Very poor
51–100	Plenty of problems
101–150	More positive than negative
151–200	Excellent

**Table 4:** Principal characteristics of DREEM subscales and score interpretation.

*Questions*	*Items*	*Max score*	*Interpretation*
**Students’Perception of Learning (SPL**)	12	48	0–12 Very Poor
			13–24 Teaching is viewed negatively
			25–36 A more positive perception
			37–48 Teaching highly thought of
**Students’Perception of Teachers (SPT)**	11	44	0–11 Abysmal
			12–22 In need of some retraining
			23–33 Moving in the right direction
			34–44 Model Teachers
**Students’Academic Self-Perceptions (SASP)**	8	32	0–8 Feelings of total failure
			9–16 Many negative aspects
			17–24 Feeling more on the positive side
			25–32 Confident
**Students’Perception of Atmosphere (SPA)**	12	48	0–12 A terrible environment
			13–24 There are many issues which need changing
			25–36 A more positive atmosphere
			37–48 A good feeling overall
**Students’Social Self-Perceptions (SSSP)**	7	28	0–7 Miserable
			8–14 Not a nice place
			15–21 Not too bad
			22–28 Very good socially

**Fig 2:**
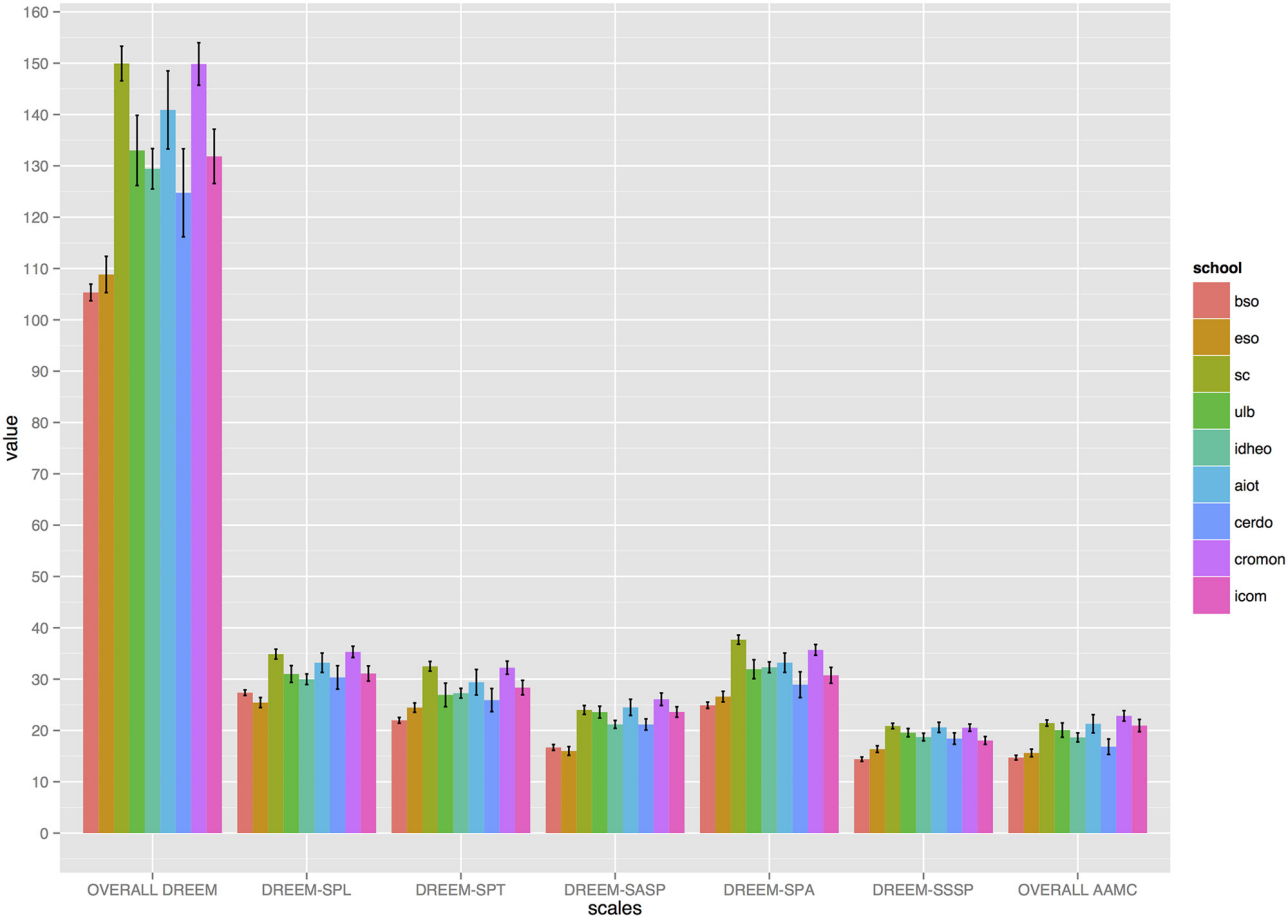
Overall AAMC, overall and subscales DREEM differences between institutions.

Fig 2 illustrates differences between institutions according to the total and subscale DREEM scores as well as the AAMC values. Detailed differences between institutions along with effect sizes are shown in S4 Table. Generally, the BSO and ESO scored significantly lower compared to all other schools in all questionnaires’ domains (p<0.001) with a large effect size between 1.0 and 3.5. Large effect sizes were demonstrated between other institutions.

### Regulated vs. not-regulated countries

**Fig 3:**
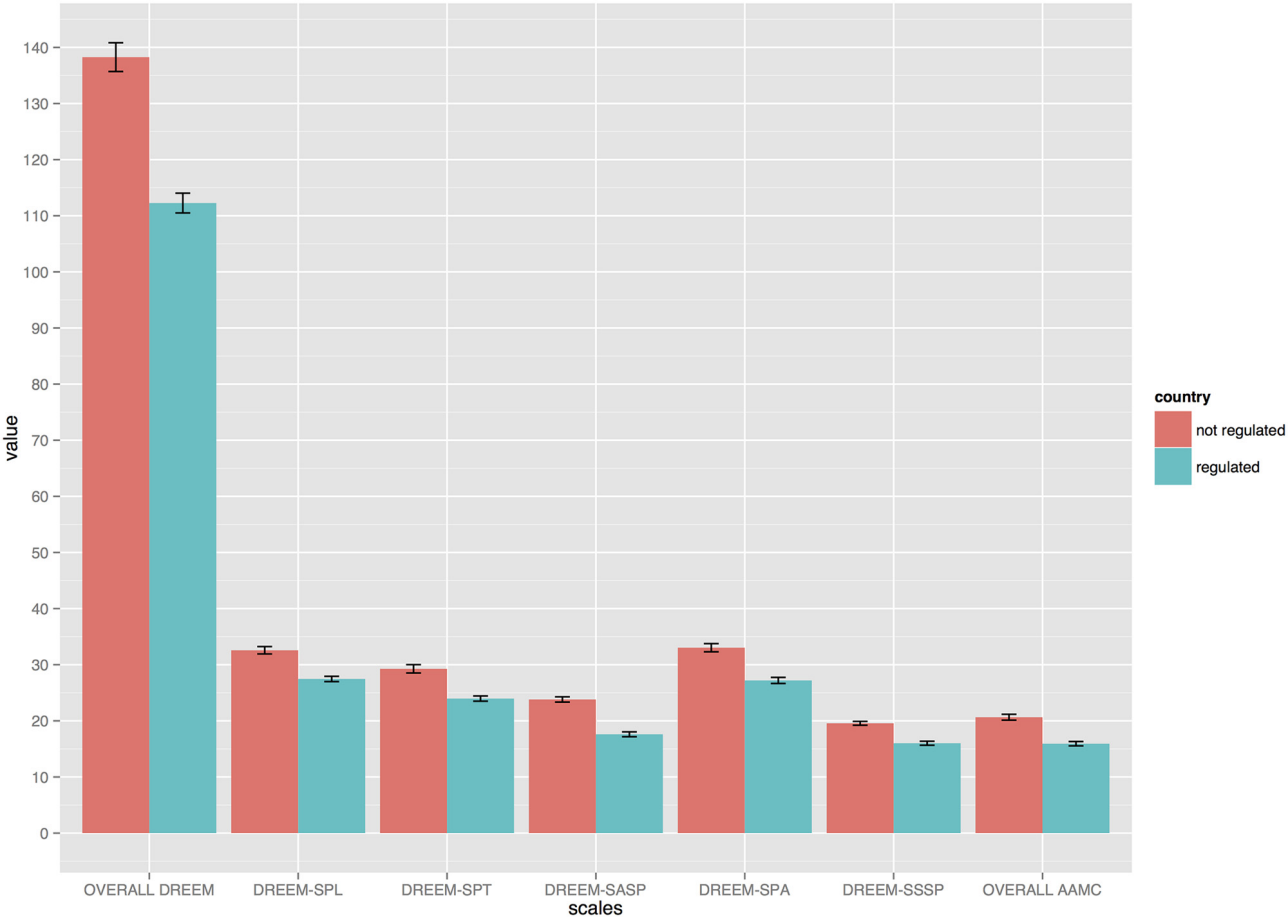
Overall AAMC, overall and subscales DREEM differences between regulated and not regulated countries.

For the purpose of analysis, institutions were divided into countries where osteopathy is or is not legally regulated (regulated vs. not-regulated countries). BSO, ESO and IdHEO were the only institutions located in regulated countries. Fig 3 shows a statistically significant difference across DREEM as well as AAMC scores between countries (p<0.001). A further analysis using a generalised linear model found a statistical association between not-regulated countries and total as well as subscales DREEM score (p<0.001; Table 5). Gender was the only other factor that was further statistically associated with a change in DREEM scores. Similar results were found if the AAMC outcome was considered (p<0.03; Table 5).

**Table 5:** Generalised linear model for questionnaire domains.

	Overall DREEM score			SPL			SPT			SASP			SPA			SSSP			AAMC		
	в	95% CI	p<|t|	в	95% CI	p<|t|	в	95% CI	p<|t|	в	95% CI	p<|t|	в	95% CI	p<|t|	в	95% CI	p<|t|	в	95% CI	p<|t|
Female	8.12	2.29; 13.96	<0.001	1.03	-0.52; 2.58	0.19	1.79	0.17; 3.40	0.03	1.77	0.44; 3.09	<0.01	2.10	0.35; 3.85	0.02	1.44	0.39; 2.49	<0.01	1.57	0.30; 2.83	<0.01
Regulated Country	-24.56	-30.65; -18.47	<0.001	-4.73	-6.35; -3.11	<0.001	-4.99	-6.68; -3.30	<0.001	-5.99	-7.37; -4.61	<0.001	-5.53	-7.36; -3.70	<0.001	-3.33	-4.42; -2.23	<0.001	-4.44	-5.76; -3.11	<0.001
Age	0.11	-1.11; 1.32	0.86	0.02	-0.31; 0.34	0.93	0.03	-0.30; 0.37	0.86	0.05	-0.23; 0.32	0.74	-0.001	-0.37; 0.36	0.99	0.02	-0.20; 0.23	0.89	-0.12	-0.39; 0.14	0.36
No previous experience	5.63	-2.65; 13.90	0.18	1.58	-0.62; 3.78	0.16	1.27	-1.01; 3.57	0.27	0.96	-0.92; 2.83	0.32	0.97	-1.51; 3.46	0.44	0.84	-0.65; 2.33	0.27	0.14	-1.66; 1.93	0.88

Education institutes in recognised countries are British School of Osteopathy, European School of Osteopathy and Institut des Hautes Etudes Ostéopathiques. SPL: Student Perception of Learning; SPT: Student Perception of Teacher; SAPS: Student academic self perception; SPA: Student Perception of Atmosphere; SSSP: Student Social Self Perception; в = Adjusted mean difference.

### Correlation between DREEM and AAMC

**Fig 4:**
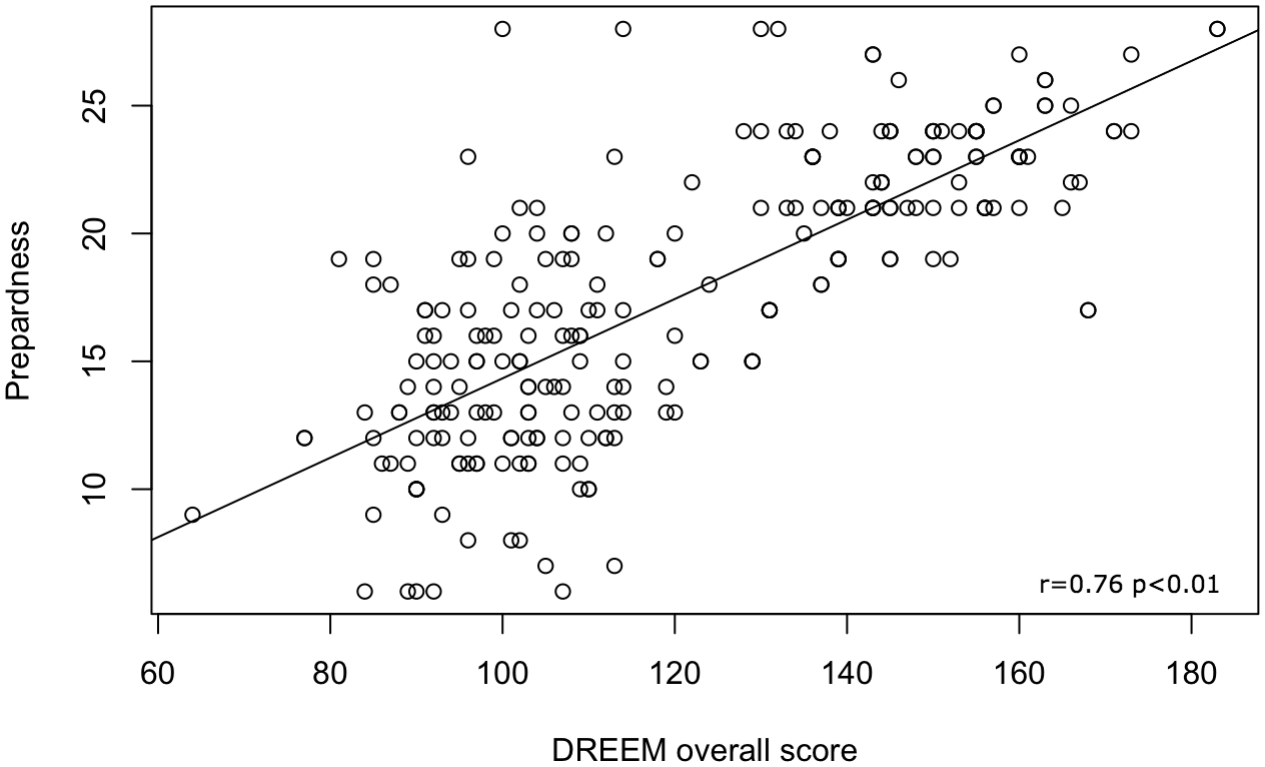
Linear correlation between DREEM and AAMC.

Fig 4 shows an overall positive linear correlation between DREEM and AAMC (r = 0.76, p<0.01). Differences in correlations were observed if a subgroup analysis by institution was performed (Table 6). Almost all the sample reached a statistically significant correlation between DREEM and AAMC (r range: 0.21 to 0.89; p-value <0.03). The only institutions that did not show significant correlations across different domains were the Sutherland College (none of the questionnaire domains), CROMON (domains: SPA: r = 0.44, p = 0.13; SSSP: r = 0.03, p = 0.96), ULB University (SSSP: r = 0.72, p = 0.07) and ICOM (SSSP: r = 0.05, p = 0.87) (Table 6).

**Table 6:** Correlation coefficients between AAMC and DREEM subscales by institution.

	SPL		SPT		SASP		SPA		SSSP	
Institutions	r	Pr(>|t|)	r	Pr(>|t|)	r	Pr(>|t|)	r	Pr(>|t|)	r	Pr(>|t|)
BSO	**0.21**	**0.039**	**0.38**	**<0.001**	**0.18**	**0.036**	**0.29**	**0.001**	**0.31**	**0.023**
ESO	**0.31**	**0.002**	**0.38**	**<0.001**	**0.66**	**<0.001**	**0.48**	**<0.001**	**0.44**	**0.005**
SC	-0.07	0.803	0.14	0.601	0.45	0.093	0.06	0.821	0.14	0.787
ULB	**0.62**	**0.001**	**0.48**	**<0.001**	**0.87**	**<0.001**	**0.49**	**0.002**	0.72	0.072
IDHEO	**0.43**	**<0.001**	**0.57**	**<0.001**	**0.85**	**<0.001**	**0.53**	**<0.001**	**0.55**	**<0.001**
AIOT	**0.83**	**<0.001**	**0.57**	**0.001**	**0.89**	**<0.001**	**0.62**	**0.005**	0.73	0.120
CERDO	**0.51**	**0.001**	**0.52**	**0.001**	**0.90**	**<0.001**	**0.41**	**0.003**	**0.49**	**0.002**
CROMON	**0.74**	**0.013**	**0.52**	**0.034**	**0.75**	**0.002**	**0.44**	**0.135**	0.03	0.958
ICOM	**0.42**	**0.003**	**0.53**	**<0.001**	**0.80**	**<0.001**	**0.46**	**<0.001**	0.05	0.865

BSO: British School of Osteopathy;ESO:European School of Osteopathy; IdHEO: Institut des Hautes Etudes Ostéopathiques; ULB: ULB University; SC:Sutherland College; ICOM: International College of Osteopathic Medicine; CROMON: Centro Ricerche Olistiche per la Medicina Osteopatica e Naturale; CERDO: Centre pour l'Etude, la Recherche et la Diffusion Osteopathiques; AIOT: Accademia Italiana Osteopatia Tradizionale.

## Discussion

The results of the present study showed that all institutions had a total DREEM score that would be classified as ‘more positive than negative’ according to McAleer and Roff’s interpretation [23]. Moreover, students’ perceived preparedness was acceptable. Taken together, these findings would suggest that there are areas of the osteopathic curriculum at each institution which merit attention for improvements. Interestingly, a higher level of students’ preparedness and satisfaction occurred in osteopathic education institutions located in countries where the profession is not regulated compared to those where regulation exist. Furthermore, in general, osteopathy student cohorts with fewer than 20 students scored significantly higher compared to larger cohorts, this may be attributed to a better interaction between students as well as between students and teachers [24]. Finally, an overall positive correlation between students’ level of preparedness and satisfaction with learning environment was found across all institutions recruited. Several hypotheses can be drawn to interpret and explain these findings.

### Differences between regulated vs not-regulated countries

Among the institutions included, some of them were located in countries where osteopathy is regulated. This could have produced a potential ‘regulation effect’ in the present study. This can be explained if the different European situation is taking into account (S1 File. Differences between countries).

The European situation, is widely heterogeneous in terms of length of full-time programmes (four to six years), type of training methods (problem based learning vs traditional), national standards and academic procedures. This can create a possible different impact on osteopathy students which, in turn, can reflect several business and educational attitudes and scenarios settled by each institution.

It should be pointed out that the ‘regulating effect’ has been possibly biased by a ‘British effect’, where the BSO and the ESO weighted more, compared to IdHEO, on the final pooled scores. This is confirmed by the statistically significant differences between the British institutions and the French one (see S4 Table). Possible interpretations of these results, which one may consider include: the larger sample size enrolled in UK compared to France, the different academic standards and, according to our data, the differences in Students’ Academic Self-Perceptions. The latter was the most important factor accounting for differences among institutions, therefore, we could speculate that the educational environment should have significantly accounted for these differences. In particular, one can argue that although the current pre-registration advanced undergraduate and postgraduate nature of osteopathic education in the UK prepares students to critically appraise available evidence and professional knowledge; it does nonetheless raise their expectations and make them more critically aware of deficiencies in their education. Therefore, students may be more likely to critically reflect on their existing competence profile and consider it inadequate for autonomous clinical practice.

Even though one can fairly assume that this attitude of critical appraisal is just as present in a six year public university programme (ULB, Belgium), the ULB-students score significantly better at Students’ Academic Self-Perception, arguing that still other factors might be involved. Considering the peculiar situation of osteopathy in Belgium of being regulated as a healthcare profession by a law which has still not been implemented after 15 years, one can argue that socio-political influences might be involved in the way students think about their professional identity, education and competence profile [25]. This is especially true when the osteopathic profession, in their communication with society and policy makers, is academically represented by a rigorous evidence based education programme that is somehow politically used to strive for regulation on the one hand and four non-accredited private education institutions considering themselves as the true defenders of the original osteopathic profession, on the other hand. Moreover, the smaller sample size in the case of the ULB-students as a possible important factor to account for has to be attenuated, because of several courses being offered in a truncus communis education programme together with physical therapy students in their bachelor degree programme and with medical students in the master degree programme.

The small class size is one of the common factors that has been showed in not-regulated private institutions in Italy and the Netherlands, which performed significantly higher on the overall and subscales, including Students’ Academic Self-Perception, DREEM scores. Several speculations could be argued to justify these findings according to: the nature of the institution (private small-medium enterprises) within a not-regulated national situation, marketing strategies, competitions which possibly influence the nature of the service delivered, attitude of the institution staff in regards of students and attention of the school system towards the client (student). In a competitive private market, where the quality (of education) is essential and the need to create new professions is critical, student satisfaction is one of the core outcomes to be achieved. Therefore, although the statutory regulatory process is largely at its early stages, the “education marketing” is organising by itself producing better services but the overall quality remains an aspect to be explored.

### Comparison with previous studies

Comparing the mean total DREEM score of final year students to those reported in previous studies [16, 17], similar results were revealed. Whereas Luciani et al. (2014) reported a DREEM score of 136; Vaughan et al. (2014) mean score was 133. Although in the present study the mean score was 121, thus lower compared to Luciani et al. (2014) and Vaughan et al. (2014), data can be interpreted as ‘a more positive than negative’ osteopathy programmes. It should be pointed out that both Luciani et al. (2014) and Vaughan et al. (2014) studies enrolled a sample smaller than the present research, although Luciani et al. (2014) recruited a population more similar in terms of geographical and geopolitical situation.

In the present study, within-institution differences for BSO and AIOT were showed comparing the 2011 and 2014 surveys. Consistent imbalances were revealed for the BSO, where the overall DREEM score lowered about 25 points in 2014 and subscale DREEM scores decreased, on average, by 4 points. Lower differences were showed for AIOT (2014–2011: overall -8 points, subscales, on average, -1 point).

Interestingly, a ‘gender effect’ was shown in the present study and these results are consistent with other studies [13, 16]) but not with Vaughan et al. (2014). To date, no formal explanation can be drawn to interpret this result as robust and reliable findings are still elusive. Therefore, further investigations should be planned to deeply explore this finding, especially in light of the ongoing global feminization of the osteopathic profession [26–27].

Considering the students’ perceived preparedness (AAMC questionnaire), the score obtained by this large European sample was lower compared to previous research [16] (17.5 vs 22.3). This could be interpreted as a decrease of perception of preparedness between final year students in 2011 and 2014, however this is particularly significant for the BSO where a decrease of almost 5 points has been shown.

### Impact

Interestingly, the DREEM domains can suggest different actions to be taken into account for improving institutional programme and, therefore, targeting the curriculum onto specific aspects. Moreover, comparing different institutions from different European countries, can produce baseline data to systematically benchmark yearly data of any institution within European trends. This can have several impacts on the policy, strategies and actions of institutions both between countries but also within the same country. Particularly, if the students’ educational and professional mobility is considered as a key topic of international development. Thus, up-to-date data on the institutional profile in terms of preparedness to practice and satisfaction can be used to tailor educational as well as marketing plans. Furthermore, the correct use of this data can be useful for institutions to develop systematic and robust mechanisms of clinical effectiveness correlated to preparedness to practice. This can have two level of interests: at institutional level (i.e., during the years of practice and tutoring) and after graduation (i.e., when new osteopaths set their own private practice). As a feed-forward mechanism, this can produce several key information for the institution in terms of predictors of clinical effectiveness as well as being used as a proxy for optimising the provision of pre-registration clinical education in osteopathy.

### Strengths and limitations

The present research included the largest cohort of European students from 9 different institutions located in 5 different European countries leading to a fairly representation of osteopathy in Europe. Moreover, the students’ response rate was high suggesting a generalisability of results within the included institutions. Notwithstanding these strengths, several limitations have to be outlined. Despite these findings, studies found that self-assessment has limited inter- and intra-validity [28, 29] (i.e., truthfulness of students’ answers); students’ satisfaction is possibly limited to final-year semesters (when the survey was administered) and not to the curriculum as a whole; findings for the Sutherland's College were possibly limited to the English version of the questionnaire although the teaching course is in Dutch. Furthermore, although internal consistency has been shown, administering the same questionnaires but in different languages could have biased the current results. Additionally, sample differences would have possibly limited a full comparison between institutions.

### The way forward

Further studies could be planned to evaluate rigorously the differences between public (university-based) vs. private education and training programmes and trends in all osteopathy training students from enrolment to final year. Moreover, including a larger sample of osteopathic institutions in Europe and not only could increase the generalisability of results as well as could serve as a proxy for policymakers and stakeholders. Certainly, a survey one year after the end of the study could be indicative of several aspects of professional onset.

## Conclusion

The present study showed a perceived higher level of preparedness and satisfaction amongst students from osteopathic institutions located in countries without regulation, compared to those located in countries where osteopathy is regulated; however, all institutions obtained a ‘more positive than negative’ result. Moreover, it demonstrated that, in general, cohorts with fewer than 20 students scored significantly higher compared to larger student cohorts. Finally, an overall positive correlation between students’ preparedness and satisfaction with the learning environment were found across all institutions recruited.

## Supporting Information

S1 File Differences between countries. (DOCX)

S1 Table Total number of participants by country and institution. (PDF)

S2 Table Total and subscales DREEM scores validity. SPL: Student Perception of Learning; SPT: Student Perception of Teacher; SASP: Student academic self-perception; SPA: Student Perception of Atmosphere; SSSP: Student Social Self Perception.
(PDF)

S3 Table Item-to-total scores for DREEM questionnaire. SPL: Student Perception of Learning; SPT: Student Perception of Teacher; SASP: Student academic self-perception; SPA: Student Perception of Atmosphere; SSSP: Student Social Self Perception.
(PDF)

S4 Table Overall DREEM, overall AAMC and DREEM subscales differences between each institutions. (XLSX)
