# Supplementary material for: Learning Environment, Preparedness and Satisfaction in Osteopathy in Europe: The PreSS Study
Source: PLoS One. 2015 Jun 23;10(6):e0129904. doi: 10.1371/journal.pone.0129904 (PMC4477891; doi:10.1371/journal.pone.0129904)
Supplement: S1 File — (DOCX) [file pone.0129904.s001.docx]

In the UK, osteopathy is statutory regulated by the General Osteopathic Council (GOsC) and there are 11 higher education institutions providing pre-registration education training courses [1]. Students completeattend a minimum of at least 1,000 hours of superevised clinical practice and follow a four-year full-time or five-year part-time course, obtaining a B.Sc. (Hons), B.Ost. or Master Degree in osteopathy (M.Ost.) depending on the school attended. Moreover, medical practitioners and physiotherapists are able to follow a 2 year full-time course (completing attending 1,000 hours of clinical education hours) to obtain an MSc in Osteopathy (pre-registration). Non-UK qualified osteopaths are able to join the GOsC following an assessment of their competence profile [2].

In Italy, osteopathy is not regulated, the quality of the teaching is controlled by the Registro degli Osteopati d'Italia (ROI – National Italian Register) in 24 schools, among them 19 have also full-time programme [3]. Students are required to should attend a minimum of t least 1,000 hours (full-time programmesclasses) or 150 hours (part-time programmesclasses) of supervised osteopathic clinical practice to graduate [4]. Graduates obtain a DO - Diploma in Osteopathy. Since 1989, ROI is playing a key role for the statutory regulation of osteopathy in Italy.

In Belgium, although a legislation (Colla Law) on non-conventional medical practices, which including es osteopathy, exists was passed since 1999, theis law has not yet been implemented.[5] In 2012, The Chamber of Osteopathy, a formal body of the Ministry of Health, decided in 2012 that the level of training in osteopathy should be at master level [6].Currently, tThere are five education institutions offering training in osteopathy, four of which are private and only one officially recognised full-time university programme. Students, of the six-year full-time university programme, graduate with a master in motor sciences (osteopathy department) and a master in clinical osteopathy and have attended 1,380 hours of clinical practice. Students of the private institutions follow a four to six year part- or full-time course and three out of four offer the opportunity to obtain a master degree in collaboration with a higher education institute in another countryabroad [5].

In France, osteopathy has been regulated since 2002. The first accreditation occurred in 2007 by the French Government. Currently, there are 30 schools providing full-time education, which ranges from five to six years in duration. There are 45 part-time courses, with variable length, the only requirement being in the total number of hours, which varies depending on the previous degree qualification [7, 8]. At the time of this study, the minimum required number of clinical education hours to be attended to graduate as an osteopath was not specified by the law. Last year, students of IdHEO were approaching 1200 hours when the questionnaires were sent. In December 2014, a new law set the number of clinical hours at 1,500 hours (full time course)  and 150 validated consultations (both full time and part time courses). Students graduate with a Diploma in Osteopathy (DO). There is no single statutory regulatory body for osteopathy in France.

In the Netherlands, osteopathy is not regulated as a distinct profession and is not protected by law [9]. Osteopathic training is offered by three private institutions that are registered and controlled by the Dutch Register of Osteopaths (Nederlands Register voor Osteopathie - NRO). Students follow a four to six year part- or full- time course and complete attend a minimum of 500 (part-time course) or 1,200 clinical education hours (full-time course) [10]; students. They  graduate with a Diploma in Osteopathy (DO).

1. General Osteopathic Council. Training Courses. 2015. Available: <http://www.osteopathy.org.uk/training-and-registration/becoming-an-osteopath/training-courses/>.
2. 26. GOsC. Qualified outside the UK*.* 2015. Available: <http://www.osteopathy.org.uk/practice/How-to-register-with-the-GOsC/Qualified-outside-the-UK/>.
3. 27. Registro degl Osteopati d'Italia. Formazione. 2014. Available: <http://www.registro-osteopati-italia.com/it/formazione/scuole>.
4. 28. ROI. Regolamento didattica*.* 2015. Available: <http://www.registro-osteopati-italia.com/ui/download/REGOLAMENTO-COMMISSIONE-DIDATTICA-2012-revisione-26_11_131.pdf>.
5. 29. van Dun PLS, Mens M, Van den Berghe W, Hermans B. Informatiedocument Osteopathie, Brussel, 2013, Groepering Nationaal en Representatief van de Professionele Osteopaten vzw (GNRPO vzw) en Register voor de Osteopaten van België (R.O.B.)*.* 2013.
6. 30. Federale overheidsdienst Volksgezondheid. Veiligheid van de Voedselketen en Leefmilieu, Kamer Osteopathie/2012/Advies K3. 2012. Available: <http://health.belgium.be/internet2Prd/groups/public/@public/@dg2/@healthprofessions/documents/ie2divers/19083674.pdf>.
7. 31. Ministère des Affaires sociales. Liste des établissements de formation en ostéopathie agréés par le ministère chargé de la santé jusqu'au 31 août 2015. 2014. Available: <http://www.sante.gouv.fr/IMG/pdf/Liste_des_etablissements_agrees_jusqu_au_31_aout_2015.pdf>.
8. 32. Syndicat Français des Ostéopathes. liste ecoles osteopathie. 2015. Available: <http://www.osteopathe-syndicat.fr/etudiant-en-osteopathie/liste-ecoles-osteopathie.html>.
9. 33. FORE. Osteopathy in the Netherlands. 2015. Available: <http://www.forewards.eu/members/our-members/netherlands/>.
10. 34. College van Advies. Beroepscompetentieprofiel Osteopathie, Nederlandse Vereniging voor Osteopathie, Hilversum en Stichting het Nederlands Register voor Osteopathie. 2009. Available: <http://osteopathie-nro.nl/media/criteria_en_reglementen/beroepscompetentieprofiel.pdf>
