# Supplementary material for: Learning Environment, Preparedness and Satisfaction in Osteopathy in Europe: The PreSS Study
Source: PLoS One. 2015 Jun 23;10(6):e0129904. doi: 10.1371/journal.pone.0129904 (PMC4477891; doi:10.1371/journal.pone.0129904)
Supplement: S1 Table — (PDF) [file pone.0129904.s002.pdf]

Name	N responders	N total	ratio
**Total**	**243**	**314**	**0,77**
Italy	**55**	**64**	**0,86**
AIOT	10	10	1,00
CERDO	12	15	0,80
CROMON	13	14	0,93
ICOM	20	25	0,80
**France**	**39**	**61**	**0,64**
IdHEO	39	61	0.64
**UK**	**119**	**150**	**0,79**
BSO	75	90	0,83
ESO	44	60	0,73
**Belgium**	**14**	**19**	**0,74**
University of Brussels	14	19	0,74
**The Netherlands**	**16**	**20**	**0,80**
Sutherland College	16	20	0,80
