# Supplementary material for: Learning Environment, Preparedness and Satisfaction in Osteopathy in Europe: The PreSS Study
Source: PLoS One. 2015 Jun 23;10(6):e0129904. doi: 10.1371/journal.pone.0129904 (PMC4477891; doi:10.1371/journal.pone.0129904)
Supplement: S2 Table — SPL: Student Perception of Learning; SPT: Student Perception of Teacher; SASP: Student academic self-perception; SPA: Student Perception of Atmosphere; SSSP: Student Social Self Perception. (PDF) [file pone.0129904.s003.pdf]

DREEM	raw alpha	std alpha
**Total**	0.90	0.90
**SPL**	0.77	0.92
**SPT**	0.77	0.93
**SASP**	0.79	0.93
**SPA**	0.76	0.92
**SSSP**	0.80	0.94
