# Supplementary material for: Learning Environment, Preparedness and Satisfaction in Osteopathy in Europe: The PreSS Study
Source: PLoS One. 2015 Jun 23;10(6):e0129904. doi: 10.1371/journal.pone.0129904 (PMC4477891; doi:10.1371/journal.pone.0129904)
Supplement: S3 Table — SPL: Student Perception of Learning; SPT: Student Perception of Teacher; SASP: Student academic self-perception; SPA: Student Perception of Atmosphere; SSSP: Student Social Self Perception. (PDF) [file pone.0129904.s004.pdf]

**DREEM**	**r**	**r cor**	**r drop**
**total**	1.00	1.00	1.00
**SPL**	0.87	0.87	0.84
**SPT**	0.86	0.86	0.83
**SASP**	0.81	0.81	0.77
**SPA**	0.89	0.89	0.86
**SSSP**	0.78	0.78	0.72
